# Managing anticoagulation in the COVID-19 era between lockdown and reopening phases

**DOI:** 10.1007/s11739-020-02391-3

**Published:** 2020-06-08

**Authors:** Daniela Poli, Alberto Tosetto, Gulatiero Palareti, Doris Barcellona, Antonio Ciampa, Elvira Grandone, Cesare Manotti, Marco Moia, Alessandro Squizzato, Vincenzo Toschi, Sophie Testa, On the behalf of Italian Federation of Anticoagulation Clinics (FCSA)

**Affiliations:** 1 Dipartimento Cardiotoracovascolare, Centro Trombosi, Azienda Ospedaliero-Universitaria Careggi, Florence, Italy; 2 UOS Centro Malattie Emorragiche e Trombotiche - Divisione di Ematologia, Ospedale S. Bortolo, Vicenza, Italy; 3 Fondazione Arianna Anticoagulazione, Bologna, Italy; 4 Dipartimento di Scienze Mediche e Sanità Pubblica, Università di Cagliari, Cagliari, Italy; 5 Centro Emostasi, AORN S.G. Moscati, Avellino, Italy; 6 Thrombosis and Haemostasis Unit, Fondazione IRCCS “Casa Sollievo della Sofferenza”, San Giovanni Rotondo, Italy; 7 Ob/Gyn Department of The First I.M. Sechenov Moscow State Medical University, Moscow, Russia; 8 Centro Emostasi, Medicina Interna ad indirizzo angiologico e coagulativo, Dipartimento di Medicina Interna e Specialistica, Azienda Ospedaliero-Universitaria di Parma, Parma, Italy; 9 IRCCS MultiMedica Group, Milan, Italy; 10 Department of Medicine and Surgery, Research Centre on Thromboembolic Diseases and Antithrombotic Therapies, University of Insubria, Varese and Como, Italy; 11 Department of Hematology and Blood Transfusion, ASST Santi Paolo e Carlo, Milan, Italy; 12 Centro Emostasi e Trombosi, Ospedale di Cremona, Cremona, Italy

**Keywords:** Oral anticoagulants, Warfarin, DOAC, COVID-19, Pandemic

## Abstract

Patients on anticoagulant treatment are constantly increasing, with an estimated prevalence in Italy of 2% of the total population. The recent spreadout of the COVID-19 pandemic requires a re-organization of Anticoagulation Clinics to prevent person-to-person viral diffusion and continue to offer the highest possible quality of assistance to patients. In this paper, based on the Italian Federation of Anticoagulation Clinics statements, we offer some advice aimed at improving patient care during COVID-19 pandemic, with particular regard to the lockdown and reopening periods. We give practical guidance regarding the following points: (1) re-thinking the AC organization, (2) managing patients on anticoagulants when they become infected by the virus, (3) managing anticoagulation surveillance in non-infected patients during the lockdown period, and (4) organizing the activities during the reopening phases.

## Introduction

Patients on anticoagulant treatment are constantly increasing, with a prevalence of nearly 2% of the total population [1].

Anticoagulation Clinics (ACs) routinely manage thousands of patients taking anticoagulants, either vitamin K antagonists (VKAs), direct oral anticoagulants (DOACs), or parenteral drugs. ACs are pivotal in anticoagulation management, providing site visits for patients, remote monitoring, and integration with local health facilities [2].The recent spreadout of the COVID-19 pandemic requires a re-organization also of ACs, to prevent person-to-person diffusion. As most of the patients taking oral anticoagulants show at least one comorbid condition, it is becoming more evident that the ACs should continue to offer the highest possible quality of assistance, while assuring all safety standards against viral transmission.

In this paper, based on the Italian Federation of Anticoagulation Clinics (FCSA) statements, we offer some advice aimed at improving patient care during COVID-19 pandemic, with particular regard to the lockdown and reopening periods. We give practical guidance regarding the following points (Table 1):

**Table 1:** Managing Anticoagulation Clinics during COVID-19 pandemic: suggested actions

	Actions
The AC organization	The centers should remain open Act as hospital consultant in the management of COVID-19 anticoagulated patients. Assure protective devices available Measure body temperature before staff and patients enter into the clinic Personnel and patients must wear masks and wash hands with hydroalcoholic solutions Organize a strict appointments agenda Favor the use of portable monitors Patients should have home drug supplies for a long time (nearly 3 months) Facilitate PT INR controls in home patients, through nurses and patient’s association Improve communications between patients and AC doctors through phone calls and/or e mail
Management of patients on anticoagulants with COVID-19 infection	In hospitalized patient or in patients treated with antiviral drugs switch from oral to parenteral anticoagulants
During the lockdown period	Re-consider the prolongation of INR controls between 4 and 8 weeks Favor telemedicine services For patients on DOAC delay visits providing telephone or email contacts
During the reopening phases	Strictly maintain all precautions recommended for ensure security for staff and patients Rapidly active clinical and laboratory control for all patients For AVK patients: organize PT-INR control for patients not reached during the lockdown phase Switch from AVK to DOAC if possible
In all phases	Participate to local and national researches

1. Re-thinking the AC organization.
2. Managing patients on anticoagulants when they become infected by the virus.
3. Managing anticoagulation surveillance in non-infected patients during the lockdown period.
4. Organizing the activities during the reopening phases.

### Re-thinking the AC organization

During pandemic periods and social distancing, all non-urgent health activities, such as periodical control visits in patients with chronic diseases or elective surgery, have been interrupted. Due to the necessity to maintain periodic controls in all anticoagulated patients, we suggests the following:

1. The AC ambulatory service should remain open. The staff (physicians, nurses, and administrative personnel) should routinely wear surgical masks and perform frequently hand hygiene.
2. Access to the AC should be verified and redesigned if necessary. Each patient must wear masks and wash hands with hydroalcoholic solutions before accessing the AC. Also, patients should be checked for fever and exposure to potential COVID-19 cases. Patients with possible exposure to COVID-19 cases or with body temperature > 37.5 °C should not be allowed to enter the center and immediately referred to the COVID-19 fast-track service [3].
3. Only the patient, except for a single caregiver in very special clinical situation (cognitive or motor limitations), should access the AC. Allowance for at least 1.5 m between patients should be provided in the waiting room.
4. For those patients on VKAs, the use of portable monitors to check PT INR should be recommended and rapid training performed.
5. All patients treated with either VKAs, DOACs, or heparin should be informed to keep at home adequate drug supplies (nearly 3 months).
6. Direct, accessible communication channels should be made available to the patients and/or their caregivers (e.g., by phone calls or e-mail).
7. Information material should be made available, aiming to address ordinary difficulties in the routine management of the treatment (e.g., minor bleedings and drug–drug interactions).

### Managing patients on anticoagulants when they become infected by the virus

#### Asymptomatic or paucisymptomatic patients

Many COVID-19 patients may be treated and discharged to home. For these patients, continuing the usual treatment dose should be recommended, conditional that the patient is able to maintain adequate hydration and does not need to use of interfering drugs (see below). VKAs’ treatment should be continued in patients with mechanical heart valves when a careful INR control is achievable.

#### Symptomatic patients with moderate-to-severe disease

If the clinical conditions deteriorate, several potentially important issues may cohesist. First, the concomitant use of interfering drugs may be necessary For instance, lopinavir/ritonavir may have the potential for CYP2C9/CYP3A4 induction, as well as for P-gp inhibition [4, 5]. Second, a severe coagulopathy has been widely described [6, 7]. Prolonged prothrombin time is frequently reported, making difficult the monitoring of anticoagulation with PT-INR. Third, adequate oral food intake may be impossible in these patients with unpredictable effects on the absorption of drugs. We suggest withholding oral anticoagulation with either DOACs or VKAs and start antithrombotic treatment at therapeutic dose with low-molecular-weight heparin (LMWH) in these patients. In patients with severely reduced renal function (estimated glomerular filtration rate 15–30 mL/min), we recommend measuring anti-Xa activity to maintain the therapeutic range (aXa = 0.5–1.2 UI/mL). In case of CrCL < 15 mL/min, we recommend switching to unfractioned heparin (UFH) with a target aPTT ratio of 1.5–2.0 or aXa activity 0.3–0.7 UI/mL [8].

### Manage anticoagulation surveillance in non-infected patients during the lockdown period

In this phase, the vast majority of patients are afraid of getting infected and may have difficulties in reaching the AC. Moreover, the AC should provide a support to patient with counseling through phone calls and e-mail and to physicians directly involved in the management of COVID-19 patients for optimal antithrombotic strategy.

#### Patients on VKAs

All therapeutic prescriptions should be re-considered to reduce as much as possible the number of INR controls. All patients with stable anticoagulation should receive written prescriptions for longer intervals, possibly 6–8 weeks [9, 10]. When longer intervals between laboratory controls are requested, patients should have easy access to telephone call with the doctors of AC, if new clinical problems occur.

#### Patients on DOACs

Routine control visits for patients on DOACs should be delayed, providing telephone contact to evaluate the patient’s clinical conditions and to identify selected patients who need a more careful surveillance.

### Organizing the activities during the reopening phases

Currently, the evolution of the COVID-19 pandemic is still unknown. Italy, like many other European countries and the US, are experiencing the reopening phases of real-world activities, but recurrences of COVID-19 infections are expected. In this phase, we recommend that all ACs actively follow up anticoagulated patients through laboratory and clinical controls.

#### General measures

ACs should evaluate protocols and strategies to implement telemedicine services to reduce physical contacts in the case of COVID-19 recurrences. Clinical and laboratory control should be performed in all patients and the anamnesis updated, paying particular attention to recent co-morbidities and new therapies. All centers are invited to actively participate in drafting hospital protocols, collaborating with all the staff involved in the management of COVID-19 patients.

#### Patients on VKAs

PT INR should be checked as soon as possible. In these periods, the switch from VKAs should promptly be considered, in the absence of contraindication, in all patients.

### Scientific research

All AC should participate in scientific studies that are now approved by National Ethical Committee and ongoing in our country on this new devastating disease. FCSA is always involved in scientific research and is now promoting the national START-COVID-19 registry within the frame of the wider START2-REGISTER study (ClinicalTrials.gov Identifier NCT02219984). All ACs, which are involved in the treatment of COVID-19 patients or are connected with COVID-19-dedicated hospital units, are invited to take active part in the START-COVID-19 registry (online information available).
